# Reversal and resumption of anticoagulants in patients with anticoagulant-associated intracerebral hemorrhage

**DOI:** 10.1186/s40001-024-01816-5

**Published:** 2024-04-24

**Authors:** Jingfei Yang, Jie Jing, Shiling Chen, Xia Liu, Jiahui Wang, Chao Pan, Zhouping Tang

**Affiliations:** Department of Neurology, Tongji Hospital, Tongji Medical College, Huazhong University of Science and Technology, Wuhan, 430030 Hubei China

**Keywords:** Intracerebral hemorrhage, Anticoagulants, Reversal, Resumption

## Abstract

The use of anticoagulants has become more frequent due to the progressive aging population and increased thromboembolic events. Consequently, the proportion of anticoagulant-associated intracerebral hemorrhage (AAICH) in stroke patients is gradually increasing. Compared with intracerebral hemorrhage (ICH) patients without coagulopathy, patients with AAICH may have larger hematomas, worse prognoses, and higher mortality. Given the need for anticoagulant reversal and resumption, the management of AAICH differs from that of conventional medical or surgical treatments for ICH, and it is more specific. Understanding the pharmacology of anticoagulants and identifying agents that can reverse their effects in the early stages are crucial for treating life-threatening AAICH. When patients transition beyond the acute phase and their vital signs stabilize, it is important to consider resuming anticoagulants at the right time to prevent the occurrence of further thromboembolism. However, the timing and strategy for reversing and resuming anticoagulants are still in a dilemma. Herein, we summarize the important clinical studies, reviews, and related guidelines published in the past few years that focus on the reversal and resumption of anticoagulants in AAICH patients to help implement decisive diagnosis and treatment strategies in the clinical setting.

## Introduction

Anticoagulants, which are effective for the prevention and treatment of venous thromboembolism (VTE) and the reduction of stroke risk in patients with atrial fibrillation (AF) [1], have increased in usage over the last decade [2]. Warfarin is the most commonly used Vitamin K antagonist (VKA) for preventing and treating arterial thromboembolism and VTE [3]. Direct oral anticoagulants (DOACs) include direct thrombin (factor IIa) inhibitors such as dabigatran and factor Xa inhibitors (FXa-Is) such as rivaroxaban, edoxaban, apixaban, and betrixaban [3, 4]. Moreover, heparin and its derivatives, including unfractionated heparin (UFH), low-molecular-weight heparins (LMWHs, such as dalteparin and enoxaparin), and the synthetic pentasaccharide fondaparinux, can be used for anticoagulation.

With an increasing number of patients treated with anticoagulants, the use of anticoagulants is becoming a more common cause of intracerebral hemorrhage (ICH) [2, 5]. As the second most common and deadliest subtype of stroke, ICH is defined as a brain injury caused by acute blood infiltration into the brain parenchyma, with a high mortality rate and poor prognosis [6, 7]. Anticoagulant-associated ICH (AAICH) patients have increased hematoma volumes, higher risks of secondary hematoma expansion (HE), and increased morbidity and mortality compared to ICH patients without coagulopathy [7–11]. Although AAICH can be devastating, the rapid and early reversal of anticoagulants may limit HE and improve clinical prognosis [8, 12]. However, in patients with AAICH, thrombotic events caused by discontinuing and reversing anticoagulant therapy may increase morbidity and mortality [13, 14]. The risk of recurrent ICH due to resuming anticoagulants is also a significant clinical challenge [13, 14]. Clinicians should carefully select reversal agents of VKAs, DOACs, or heparins and resume anticoagulant therapy at the appropriate times following ICH. It is necessary to formulate individualized treatment strategies for patients with AAICH. In this article, we describe the characteristics of AAICH and review the reversal and resumption of anticoagulants for the management of thrombotic or bleeding events in patients with AAICH.

## Characteristics of AAICH

The use of anticoagulants is linked to larger hematoma volumes, increased rates of HE, and contributes to an even higher mortality rate in patients with AAICH compared with general ICH patients [5, 8–10, 15]. The patients with ICH who are treated with anticoagulants have worse outcomes and higher prehospital blood pressure (BP) compared to those not taking anticoagulants [16]. In addition, patients with AAICH exhibit a higher incidence of intraventricular hemorrhage (IVH), AF, and prior stroke compared to those receiving non-antithrombotic therapy [12, 17]. Studies have shown that AAICH may preferentially involve the cerebellum [11] and form a fluid layer in the hematoma on magnetic resonance imaging (MRI), which represents hematocrit separation and is typical of hemorrhages associated with coagulopathy [18]. Furthermore, neuroimaging markers such as cerebral microbleeds (CMBs) and cortical superficial siderosis (cSS) are associated with the increased risk of bleeding in VKA-related ICH and DOAC-related ICH [19]. A recent clinical study has shown that the cerebrovascular small vessel disease (SVD) burden including white matter lesions (WML), lacunes, and cerebral atrophy, is associated with DOAC–ICH [20].

In addition, there may be a difference between VKA-associated ICH and DOAC-associated ICH. Many clinical trials and meta-analyses have demonstrated that DOACs can significantly reduce the risk of ICH compared with VKAs. Because of the similar or better efficacy/safety and reduced risk of ICH compared to VKAs, DOACs have been the preferred treatment for VTE/AF, replacing VKAs as the most commonly used anticoagulants worldwide [21–23]. A network meta-analysis that included 23 randomized controlled trials (RCTs) in patients with AF confirmed that patients using either DOAC had a significantly lower risk of ICH than those using warfarin [24]. Another milestone meta-analysis, which included 28 high-quality real-world observational studies, also showed similar results and highlighted the validity of DOACs (dabigatran, rivaroxaban, and apixaban) [25]. Rivaroxaban at a daily dose of 20 mg [26], edoxaban at a daily dose of 60 mg [27], and apixaban at a dose of 5 mg twice daily [28] could significantly reduce the risk of ICH compared to warfarin. In addition, a meta-analysis suggested that DOACs were related to a lower risk of traumatic ICH compared with VKAs [29]. Furthermore, a recent meta-analysis involving 82,404 patients with AF confirmed that DOACs reduced the risk of ICH by almost half compared to VKAs, with dabigatran 110 mg likely being the safest option [30]. Another recent meta-analysis, which focused on 55 RCTs, is the first pairwise meta-analysis to compare the risk of ICH between DOACs and other antithrombotic drugs. The analysis found that the risk of ICH with DOACs was generally lower than that with warfarin and similar to aspirin. However, it suggested that rivaroxaban might increase the risk of ICH [31].

Moreover, a recent study included 5984 patients and found that DOACs were associated with a lower hazard than VKAs for the composite outcome of ICH in patients with AF and recent ischemic stroke (IS) aged 85 years or older [32]. DOACs were associated with a smaller baseline hematoma volume and less neurological deficit than VKAs. The J-ASPECT study reported that fewer severe outcomes and lower mortality rates might be related to milder hemorrhages and lower frequencies of HE in DOAC-ICH patients compared with VKA-ICH patients [33]. Similarly, a retrospective cohort study has shown comparable results [17]. However, several other studies found no significant difference in the rate of HE, 90-day mortality, and functional outcomes between DOAC-ICH patients and VKA-ICH patients [34, 35]. The reasons for the differences between these findings could be related to the different study types, heterogeneous conditions of the enrolled patients, and different post-admission treatments. More standardized clinical studies are expected to be published to explore the differences between patients with DOAC-associated ICH and those with VKA-associated ICH. These studies can provide guidance for future clinical decisions.

## Reversal of anticoagulants in AAICH

Regardless of what kind of anticoagulants are used, earlier and complete reversal therapes are essential in patients with AAICH, as they potentially reduce the incidence of HE and improve clinical outcomes [12, 36]. The strategy of reversal therapy depends on the anticoagulants used, including specific or non-specific reversal (Table 1).

**Table 1:** Reversal therapies in anticoagulant-associated intracerebral hemorrhage

Type of anticoagulants	Specific agents	Reversal agents	Strategy for reversal	Cautions	Laboratory evaluation
Vitamin K antagonists	Warfarin	Vitamin K	10 mg IV	Anaphylaxis	INR
		FFP	4 U or 12 ml/kg IV	Fluid overload	
		PCCs	If INR 1.7–4, give 25 U/kg; if INR 4–6, give 35 U/kg; if INR > 6, give 50 U/kg. (targeting INR level < 1.3 within 4 h)	Thrombotic events	
Direct thrombin inhibitors	Dabigatran	Idarucizumab	Preferred drug Single 5 g/100 ml dose, repeat if needed	Headaches	APTT, TT
		Medicinal activated charcoal	50 g if DOACs are ingested < 2 h	-	
Direct factor Xa inhibitors	Apixaban; Betrixaban; Edoxaban; Rivaroxaban	Andexanet alfa	Preferred drug 800 mg bolus over 30 min, then 960 mg over 2 h if the last intake ≤ 7 h; 400 mg bolus over 15 min, then 480 mg over 2 h if the last intake > 7 h	Thrombotic events	Anti-Xa activity
		4-factor PCC	50 IU/kg IV (off-label)	Thrombotic events	
		Medicinal activated charcoal	50 g if DOACs are ingested < 2 h	-	
Heparinoids	UFH	Protamine sulfate	1 mg IV for every 100 U of heparin given in the previous 3 h (up to 50 mg in a single dose)	Bradycardia, hypotension	APTT, Anti-Xa activity

APTT, activated partial thromboplastin time; DOACs, direct oral anticoagulants; FFP, fresh frozen plasma; INR, international normalized ratio; IV, intravenous injection; PCC, prothrombin complex concentrate; TT, thrombin time; UFH, unfractionated heparin

### Reversal of VKAs

**Fig. 1:**
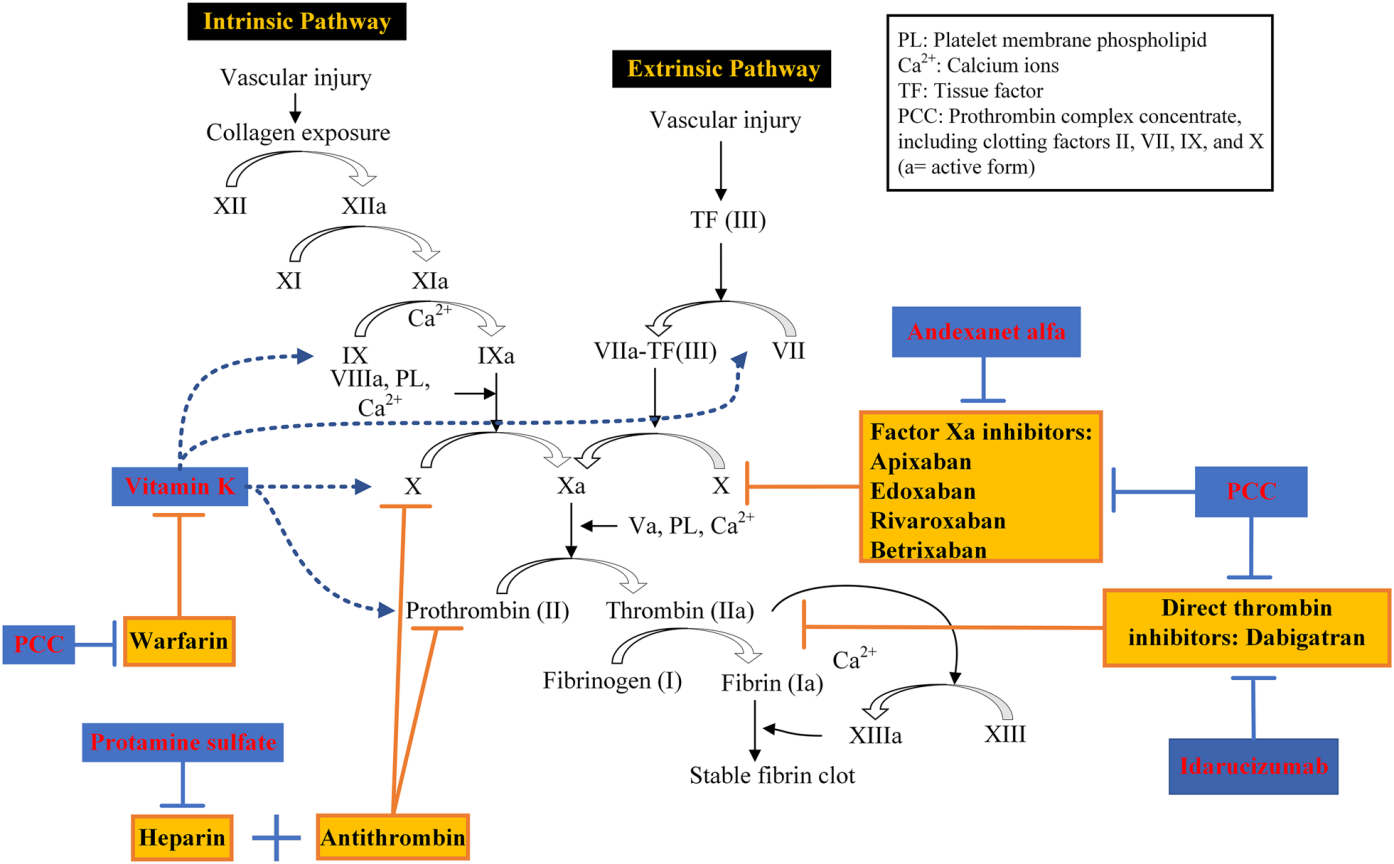
Coagulation cascade and targets of anticoagulants or potential reversal agents

Warfarin depletes vitamin K reserves, thereby disrupting the production of clotting proteins such as factors II, VII, IX, and X, as well as proteins C and S (Fig. 1) [4]. A retrospective study revealed that lower rates of HE and reduced in-hospital mortality were associated with the normalization of international normalized ratio (INR) levels to < 1.3 and systolic BP reduction to < 160 mmHg within 4 h after admission [10]. Immediate reversal therapy and strengthening the management of BP are essential to reduce HE in VKA-related ICH [12]. Vitamin K combined with prothrombin complex concentrates (PCCs) has been the primary reversal therapy for VKAs. Several studies have also investigated the use of fresh frozen plasma (FFP) or recombinant activated factor VIIa (rFVIIa) for the reversal of VKAs recently [7, 37].

Intravenous administration of 10 mg of vitamin K as the sole treatment for urgent partial reversal of warfarin for non-life-threatening bleeding may provide a sufficient hemostasis effect within 5 h [38]. However, intravenous vitamin K alone is insufficient for reversal in the case of acute ICH, because INR normalization with vitamin K takes up to a day, and most HE occurs within the first few hours of symptom onset [4, 39]. Therefore, vitamin K is usually administered in combination with FFP or PCCs to sustain a normal INR and reduce HE [40, 41]. FFP replenishes clotting factors by replacing plasma proteins and takes up to 30 h to reverse INR. However, the adverse reactions include fluid overload, which increases the risk of heart failure, transfusion-related acute lung injury, and infectious reactions [37, 40, 41].

It is recommended that PCCs be administered before FFP for urgent reversal of VKA in life-threatening major bleeding events [40, 42]. Due to the higher concentration of clotting factors in PCCs compared to FFP, data from randomized trials and large observational studies provide evidence that PCCs can rapidly reverse INR, improve hemostasis, reduce rates of HE, and lower mortality in patients with VKA-related ICH [7, 40, 43–46]. These studies supported that the use of PCCs was an effective, rapid, and appropriate treatment for the urgent reversal of VKA in patients with AAICH [7, 40, 43–46]. PCCs include 3-factor PCC (clotting factors II, IX, and X) and 4-factor PCC (clotting factors II, VII, IX, and X) [4]. The INCH trial suggested that 30 IU/kg intravenous 4-factor PCC might be superior to 20 mL/kg intravenous FFP in terms of normalizing the INR within 3 h in VKA-ICH patients. The average time for INR reversal in the PCC group was only 40 min, while in the FFP group, it was over 24 h [45]. 4-factor PCC is an effective substitute for plasma and is licensed for the rapid reversal of VKA. The dose is determined based on INR and body weight (25–50 IU/kg) [7, 44]. The latest guideline recommends using 4-factor PCC instead of FFP to achieve rapid correction of INR and limit HE in patients with VKA-related ICH and INR ≥ 2.0 [7]. However, a multicenter registry reported that PCC rapidly corrected the INR of most patients. Nevertheless, mortality and morbidity rates remained high, and discharge functional status was still poor [47]. Another study indicated that high doses of PCC (> 2000 and 3000 IU) were associated with VTE [48]. Although there is a lack of prospective RCTs to study the effectiveness and determine the optimal doses systematically, PCCs can still be recommended in VKA-associated ICH, and clinicians must carefully weigh the risks and benefits [7].

In addition, rFVIIa is a non-plasma-derived and rapid-acting potential agent for VKA-associated ICH [49]. rFVIIa at pharmacologic doses directly activates factor X on the surface of activated platelets, but it only partially replaces missing clotting factors and may not restore thrombin generation as FFP or PCC does [49, 50]. The randomized, double-blind, placebo-controlled FAST trial showed that rFVIIa significantly reduced the growth of the hematoma but failed to improve survival or functional outcome at 90 days [50]. Furthermore, later studies showed that rFVIIa increased the risk of arterial thromboembolic events and did not significantly improve radiographic or clinical outcomes in ICH patients [51, 52]. Therefore, the guideline published in 2015 recommended against using rFVIIa for the routine reversal of VKAs [37] and the latest guideline in 2022 did not mention the use of rFVIIa [7].

### Reversal of DOACs

DOACs, which have rapid antithrombotic effects, block the coagulation process by inhibiting procoagulant enzymatic activity [53]. Dabigatran, converted from dabigatran etexilate, which is an orally absorbable prodrug, is a highly selective and reversible direct thrombin inhibitor. It inhibits both free and clot-bound thrombin and prevents thrombin-induced platelet aggregation [54, 55]. Apixaban, edoxaban, rivaroxaban, and betrixaban bind to and inhibit factor Xa which is responsible for converting prothrombin to thrombin [55]. Reversal agents for DOACs include specific and non-specific drugs. The specific reversal agents include idarucizumab, a specific antagonist of dabigatran; andexanet alfa, a specific antagonist of factor Xa inhibitors; and ciraparantag, which is purported to reverse DOACs and heparins [56]. The non-specific antidotes mainly refer to PCCs, including 3-factor PCC, 4-factor PCC, and aPCC (activated VII, II, IX, X, FEIBA) [12].

#### Idarucizumab for dabigatran-associated ICH

Idarucizumab is a humanized antibody fragment that binds to dabigatran with high affinity and forms a complex, resulting in the almost irreversible reversal of anticoagulation [57]. It has a short half-life, allowing the resumption of anticoagulation within a reasonable time frame, and does not increase the risk of hypercoagulability [57, 58]. Approved by the European Medicines Agency (EMA) and the Food and Drug Administration (FDA) in 2015, this medication is used to rapidly stop dabigatran-associated bleeding complications before emergency surgery or in cases of life-threatening bleeding [59].

The full cohort analysis of the RE-VERSE AD trial, a multicenter and prospective study of 503 patients, aimed to determine whether 5 g of intravenous idarucizumab could reverse the anticoagulant effect of dabigatran in patients with uncontrolled hemorrhage or in need of an urgent procedure. In the RE-VERSE AD trial, idarucizumab was shown to rapidly, durably, and safely reverse dabigatran within minutes without serious adverse safety signals [60]. Moreover, idarucizumab has no pharmacological prothrombotic effects [58], and the thrombotic events that occurred in the study are likely associated with the underlying prothrombotic state and the failure to resume anticoagulation promptly [60]. Later, a study collected retrospective data from 61 stroke centers in German, including 27 ICH patients. The study found that idarucizumab appeared to prevent HE, improve outcomes, and reduce mortality in dabigatran-associated ICH [61]. However, there are no more detailed clinical trials or evidence regarding the effects of idarucizumab on rates of HE or clinical endpoints. The lack of imaging data limits any conclusions about clinical efficacy in DOAC-associated ICH [7].

In addition, hemodialysis has been evaluated with good results and plays a role in accelerating the elimination of dabigatran, reducing the duration and/or severity of bleeding in patients with dabigatran-associated bleeding [62–64]. Dabigatran can be reduced by hemodialysis, particularly in cases of acute kidney injury or when idarucizumab is not available [3, 7].

#### Andexanet alfa for factor Xa inhibitor-associated ICH

Andexanet alfa, a specific antagonist of direct and indirect factor Xa inhibitors, is a modified recombinant protein analog of human factor Xa that binds and sequesters factor Xa inhibitors but does not induce prothrombotic activity, thereby rapidly reversing the anticoagulant effect [65]. It was approved by the FDA in 2018 for patients treated with apixaban or rivaroxaban who have life-threatening bleeding [65].

The ANNEXA-4 trial, a large, multicenter, prospective, single-group cohort study of 352 patients, assessed the efficacy and safety of andexanet alfa in patients with acute major bleeding occurring while taking either apixaban or rivaroxaban [65]. For patients who had received apixaban or rivaroxaban more than 7 h before bolus administration, the bolus dose was 400 mg over 15 min, and the infusion dose was 480 mg. For patients who had received factor Xa inhibitors within 7 h, the bolus dose was 800 mg over 30 min, and the infusion dose was 960 mg [65]. The results indicated that 82% of patients had excellent or good hemostatic efficacy 12 h after the end of infusion, and andexanet alfa markedly reduced anti-FXa activity [65]. A recent subgroup analysis of the ANNEXA-4 trial also showed that andexanet alfa effectively reduced anti-FXa activity with a high rate of hemostatic efficacy [66]. Moreover, in a rabbit hemorrhage model, andexanet alfa effectively reversed the effects of edoxaban, indicating its clinical value in treating edoxaban-related bleeding. Anti-FXa activity could serve as a biomarker for assessing the reversal [67]. However, it is still unknown whether andexanet alfa will have greater clinical efficacy for edoxaban reversal than ciraparantag [3]. In addition, ten percent of patients in the ANNEXA-4 trial experienced thrombotic events during the 30-day follow-up period, mostly in patients whose anticoagulation therapy was delayed or not restarted [65]. Another study analyzed 182 patients from the ANNEXA-4 trial and the RETRACE-II trial, suggesting that andexanet alfa was associated with a lower rate of HE compared with PCCs. However, it did not significantly improve clinical outcomes [68]. A retrospective study also found that higher rates of hemostatic efficacy and an increased incidence of thrombosis were observed in patients with AAICH who were treated with andexanet alfa compared to 4F-PCC for the reversal of rivaroxaban or apixaban [69]. Andexanet alfa has not been widely used due to its high cost [70]. There are not enough RCTs to evaluate the safety and effects of andexanet alfa in limiting HE and preventing thrombotic events.

#### Ciraparantag

Ciraparantag (PER977) is a new synthetic cationic molecule designed to bind to direct factor-Xa inhibitors, thrombin inhibitors, and heparinoids through noncovalent hydrogen bonding and charge-charge interactions [71], resulting in the reversal of anticoagulants [3, 12, 56]. Ansell et al. found that ciraparantag (100-300 mg) administered after edoxaban restored the whole blood clotting time to baseline levels within 10-30 min, which was sustained for 24 h. In addition, the mean fibrin-fiber diameter returned to normal within 30 min [72]. In animal models of bleeding (rat tail transection and liver laceration), ciraparantag significantly reduced bleeding and blood loss induced by heparin and various DOACs. It acted rapidly, exhibited broad-spectrum activity, and was easy to administer [71]. The latest randomized, placebo-controlled Phase 2 trials suggested that ciraparantag reversed the effects of apixaban or rivaroxaban in a dose-related manner in healthy elderly individuals and was well tolerated at all doses [73]. However, this study had limitations, including the small sample size and the inclusion of healthy volunteers who could not represent patients [70]. Ciraparantag is a new potential antidote in development, with advantages that include easy and rapid preparation for injection [71, 73]. Further studies are needed to clarify the benefits and potential side effects of this drug more comprehensively.

#### PCCs

Several animal experiments, clinical studies, and meta-analyses have suggested that PCCs appear to enhance thrombin generation and nonspecifically reverse the effects of DOACs [74–80]. Studies have shown that 50 IU/kg 4-factor or 3-factor PCCs could immediately reverse the effect of rivaroxaban, as measured by prothrombin time and thrombin generation [74, 78]. A retrospective cohort study demonstrated that PCCs were associated with a high hemostasis rate (81.8%) and a low incidence of thrombotic events (3.8%) in patients with apixaban- or rivaroxaban-related ICH [79]. The UPRATE study showed that the majority of patients treated with 4-factor PCCs achieved effective bleeding control, with a low risk of serious adverse events, such as thromboembolic events [81]. There seemed to be an acceptable balance between the efficacy and safety of PCCs in patients experiencing major bleeding events related to rivaroxaban or apixaban who were administered an initial 2000 IU of PCC [81].

In addition, a large and randomized study suggested that 4-factor PCCs dose-dependently reversed the effect of edoxaban, and a dose of 50 IU/kg might be suitable for the reversal [75]. A prospective cohort study found that patients with dabigatran-related bleeding, treated with aPCCs (50 U/kg), had better outcomes and no excessive thromboembolic events compared with the control subjects [82]. A cell-based model indicated that PCC could affect thrombin generation and promote hemostasis at therapeutic dabigatran levels, and PCC normalized hemostasis time in a mouse saphenous vein bleeding model [83]. In addition, a recent meta-analysis showed that the anticoagulation reversal, mortality, or thromboembolic events appeared similar between 4-factor PCCs and andexanet alfa in the absence of randomized clinical comparison trials [84]. These studies provide valuable insight that PCCs can be a viable alternative, and 4-factor PCCs may be more supported and widely used [7].

However, other studies have demonstrated that doses of 37.5 IU/kg and 25 IU/kg of PCCs were insufficient for an immediate complete reversal of peak therapeutic levels of rivaroxaban or apixaban [76, 80]. In a new retrospective study, there was no difference in the effects of aPCCs, low- and high-dose 4-factor PCCs on hematoma stability, mortality, and safety in ICH patients taking apixaban or rivaroxaban [85]. Moreover, some studies have shown that the reversal effect of PCCs may not be effective in dabigatran-treated patients [74, 86]. PCCs failed to restore changes in fibrin formation in healthy volunteers treated with rivaroxaban or dabigatran [87]. The RETRACE II study suggested that PCC was not associated with a reduced rate of HE, mortality, or improved functional outcomes in DOAC-related ICH, failing to show any benefits [88]. Thus, large, standardized RCTs evaluating the effect of PCCs in patients with DOAC-related ICH are urgently needed in the future.

### Reversal of heparins

Heparins bind tightly to a specific antithrombin site and form a heparin-antithrombin complex, which inactivates thrombin factor (IIa) and factors Xa, IXa, XIa, and XIIa. Among these factors, thrombin and factor Xa are the most sensitive to inhibition by the complex [89]. Reversal drugs for heparins include protamine sulfate and others.

#### Protamine sulfate for reversal of UFH

Protamine sulfate is a positively charged alkaline protein extracted from fish sperm that forms a complex with negatively charged heparin by intravenous injection of 1 mg/100 U (maximum dose 50 mg), thus completely and rapidly reversing the effect of UFH [37, 77]. The activated partial thromboplastin time (APTT) can monitor the protamine-mediated reversal of UFH [77]. However, protamine sulfate may cause uncommon hypersensitivity reactions in patients with previous exposure to protamine sulfate-containing insulin, those with fish allergies, or those who have undergone vasectomy. These reactions can be pretreated with steroids and antihistamines [77, 90]. Protamine may also lead to severe adverse reactions such as hypotension, bronchoconstriction, thrombocytopenia, or bradycardia, which can be reduced by taking protamine slowly [7, 90, 91]. Therefore, the required doses of protamine sulfate need to be cautiously considered, and it is preferable to take smaller doses repeatedly [7, 92].

#### Reversal of LMWHs

LMWHs exert the anticoagulant effect mainly by inactivating factor Xa through antithrombin [77]. Protamine only partially affects the anti-Xa activity of LMWHs, and there is a need for more effective reversal agents to reverse the anticoagulant effect of LMWHs [77, 93]. Early studies found that rFVIIa could reverse the anticoagulant effects of enoxaparin and fondaparinux ex vivo, reduce LMWH-induced bleeding in rats, and was well tolerated in patients undergoing anticoagulant therapy [94–96]. However, another study suggested that rFVIIa was not an effective antidote to LMWH-related bleeding in a rabbit ear bleeding model [97]. There is currently insufficient clinical data demonstrating the reversal effect of rFVIIa in LMWH-related ICH, and the drug may be associated with undesirable clotting [98].

#### Reversal of fondaparinux

Fondaparinux does not have a specific reversal agent. Protamine has no neutralization activity against fondaparinux [37, 77]. In a randomized, placebo-controlled trial, rFVIIa (90 μg/kg) can normalize clotting times and thrombin generation and reverse the effect of fondaparinux (10 mg) when severe bleeding complications occur or urgent surgery is needed [99]. The addition of rFVIIa in vitro corrected the inhibited clot formation and partially reversed the acceleration of clot lysis induced by fondaparinux [100]. Furthermore, in a rabbit model, PCC normalized the increased clotting time and clotting formation time induced by fondaparinux and effectively reduced bleeding without increasing thrombosis [101]. In an in vitro study on the reversal of fondaparinux, low doses of aPCC at 20 U/kg can completely correct thrombin generating capacity, while rFVIIa partially corrected thrombin generation [102].

#### Other new agents

Studies have demonstrated that the universal heparin reversal agent (UHRA) can effectively bind to heparins with excellent biocompatibility, reducing heparin-induced bleeding and exceeding protamine in heparin neutralization [103]. A later study suggested that UHRA mainly formed the UHRA-heparin complex by directly and specifically binding to anionic heparin and disrupted heparin-activated antithrombin, neutralizing the activity of available heparin-based anticoagulants [104]. Moreover, a newly designed biocompatible antidote (GC4AOEG) with a strong binding affinity for UFH neutralized UFH in vitro and in vivo without adverse effects. This antidote may have important clinical potential for reversing UFH [105]. In addition, ciraparantag can weakly bind fondaparinux and bind UFH or enoxaparin with a near-micromolar affinity to reverse the anticoagulants [104]. Previously, a clinical study included 10 healthy volunteers who received incremental doses of ciraparantag (100 to 300 mg) or a placebo. The study found that ciraparantag specifically bound to enoxaparin and reversed the anticoagulant effect, as measured by the whole blood clotting time, in a dose-related manner [106]. To verify the efficacy and safety of these potential heparin reversal agents, more clinical trials, preferably RCTs, are needed in the future.

## Resumption of anticoagulants in AAICH

Due to the limited evidence, the decision to resume anticoagulants after ICH is a common clinical dilemma. Theoretically, patients with AF and AAICH are at risk of thrombosis after the reversal of anticoagulants, so anticoagulants should be restarted promptly after the patient’s condition stablizes [56]. If the bleeding is caused by secondary or reversible factors, such as trauma or a tumor, anticoagulation can generally be resumed once the bleeding cause has been resolved [21, 107]. The decision to resume anticoagulants is a topic of intense debate, because inappropriate prescription of anticoagulants may increase the risk of rebleeding. There is no clear consensus on the decision and optimal timing of restarting anticoagulants in patients with AAICH in clinical practice [108].

### Decision on anticoagulation resumption

In published clinical studies, the resumption of anticoagulant therapy in most patients with AAICH seems to provide net clinical benefits and is supported [21, 41]. Two nationwide Danish observational studies reported that the reintroduction of oral anticoagulants after ICH was associated with a significant reduction in thromboembolic events and all-cause mortality rates, without a significant increase in the risk of major bleeding [109, 110]. A retrospective cohort study including 5712 Asian patients with nonvalvular AF and prior ICH who subsequently initiated anticoagulant therapy and found that DOACs were associated with significantly reduced risks of ICH, ischemic stroke, and death compared with warfarin [111]. A cohort study published in JAMA, including 4540 patients with AF and prior ICH, also reached similar conclusions [112]. Moreover, a recent nationwide retrospective study showed that anticoagulants reduced the risk of ischemic stroke without an increase in the risk of subsequent ICH compared with no treatment. Users of DOACs also had significantly reduced all‐cause mortality compared with warfarin [113]. Several other observational and registry studies, as well as meta-analyses, also support the resumption of oral anticoagulants after ICH (Table 2) [10, 14, 114–120]. Thus, the resumption of anticoagulants is recommended in patients with AF with prior ICH, and DOACs may be a more effective and preferred treatment option for stroke prevention due to the lower risk of recurrent ICH and better functional recovery compared with VKAs [21, 121–124]. However, these studies were almost all retrospective, and there is still a lack of high-quality RCTs to guide the resumption of anticoagulants after ICH. The prospective, randomized SoSTART trial included 203 patients with ICH and AF with CHA2DS2-VASc scores of at least 2. Participants were randomly assigned to either start or avoid oral anticoagulation. The trial failed to demonstrate the benefits of resuming oral anticoagulation and showed that recurrent ICH was more frequent and fatal in the restart group compared with the avoid group [125]. In another randomized, phase 2 trial (APACHE-AF), both the apixaban group and the avoid group had high annual risks of non-fatal stroke or vascular death [126]. Overall, the high-quality evidence to guide the resumption of anticoagulants is limited, resulting in wide variation and uncertainty in clinical treatment strategies [108].

**Table 2:** Studies reporting the resumption of anticoagulants after ICH

Study (pub. year)	Design	Sample size	Resumption of anticoagulants	Time of anticoagulants restarting	Risk of hemorrhagic complications (per 100 person-years)		Risk of thromboembolism (per 100 person-years)		Outcomes of ICH patients with anticoagulants resumption
					Patients with anticoagulants resumption	Patients without anticoagulants resumption	Patients with anticoagulants resumption	Patients without anticoagulants resumption	
Suda (2023) [132]	Retrospective	160	DOACs	7 days (4–11 days)	NA	NA	NA	NA	Early resumption of DOACs after ICH appeared to be safe in patients with NVAF. Expected functional outcomes were associated with the timing of resumption
Lin (2022) [113]	Retrospective	1899	VKAs/DOACs	42 days (10–127 days)	1.4	1.6	3.5	4.9	Reduced risk of ischemic stroke, without increased risk of recurrent ICH compared with no treatment. DOACs users had lower mortality compared with warfarin
SoSTART (2021) [125]	Prospective	203	VKAs/DOACs	115 days (49–265 days)	8	4	11	22	Starting oral anticoagulation was non-inferior to avoiding it
APACHE-AF (2021) [126]	Prospective	101	DOACs (Apixaban)	46 days (21–74 days)	12	6	12.6	11.9	Starting or avoiding oral anticoagulation both had high annual risks of non-fatal stroke or vascular death
Lee (2020) [111]	Retrospective	5712	VKAs/DOACs	0.6 year (0.2–1.7 year)	NA	NA	NA	NA	DOACs use was associated with lower risks of ischemic stroke, ICH, and composite outcome than warfarin
Tsai (2020) [112]	Retrospective	4540	VKAs/DOACs	NA	NA	NA	NA	NA	DOACs use was associated with lower rates of ICH and major bleeding compared with warfarin use
Poli (2018) [114]	Retrospective	244	VKAs/DOACs	1–3 months	1.0	1.0	2.0	6.0	A lower rate of ischemic stroke/SE and all-cause mortality with no significant increase in major bleeding
Murthy (2017) [117]	Meta-analysis	5306 (8 studies)	VKAs/DOACs	A median of 10–39 days	8.7	7.8	6.7	17.6	A lower risk of thromboembolic complications and a similar risk of ICH recurrence
Chai-Adisaksopha (2017) [118]	Meta-analysis	3145 (10 studies)	VKAs (Warfarin)	31 days	6.7	7.7	3.5	7.0	Reduction of all-cause mortality and ischemic stroke and no significantly increased recurrent intracranial bleeding
Korompoki (2017) [14]	Meta-analysis	2452 (7 studies)	VKAs (Warfarin)	NA	4.6	4.0	3.2	7.3	A lower rate of ischemic stroke without causing a major increase in the risk of ICH recurrence
Nielsen (2017) [115]	Retrospective	2415	VKAs (Warfarin)	31 days	5.8	5.3	3.3	8.9	A lower rate of ischemic stroke or SE and an increased rate of recurrent ICH, but these differences did not reach statistical significance
Pennlert (2017) [116]	Retrospective	2619	NA	Within 8 weeks	6.9 per 3y	4.4 per 3y	6.3 per 3y	13.8 per 3y	A reduced rate of thrombotic events with no significantly increased rate of hemorrhagic events
Chao (2016) [122]	Retrospective	12 917	VKAs (Warfarin)	NA	5.9	4.2	3.4	5.8	The use of warfarin may be beneficial to patients who have atrial fibrillation with a previous ICH and a CHA2DS2–VASc score ≥ 6
Park (2016) [119]	Retrospective	428	VKAs (Warfarin)	117.5 ± 235.7 days	5.5	3.1	2.4	8.3	The initiation of anticoagulants at least 2 weeks after ICH was associated with improved clinical outcomes
Ottosen (2016) [110]	Retrospective	6369	VKAs/DOACs	Within first 6 months	NA	NA	NA	NA	Lower risks of all-cause mortality and thromboembolic events and no increased risk of major bleeding
Nielsen (2015) [109]	Retrospective	1752	VKAs/DOACs	34 days	8.0	8.6	5.3	10.4	A significant reduction in ischemic stroke/all-cause mortality rates
Kuramatsu (2015) [10]	Retrospective	719	VKAs or active heparinization before resumption	31 days	8.1	6.6	5.2	15.0	Lower risk of ischemic events
Witt (2015) [120]	Retrospective	160	VKAs (Warfarin)	14 days	7.6	3.7	3.7	12.3	No increased risk of recurrent ICH but trending toward reduced thrombosis and all-cause mortality

CHA2DS2–VASc, congestive heart failure, hypertension, age ≥ 75 years, type 2 diabetes, previous stroke/transient ischemic attack/thromboembolism, vascular disease, age 65 ~ 74 years, and sex category; *DOACs, *direct oral anticoagulants; *ICH*, intracerebral hemorrhage; *NA,* not available; *NVAF,* non-valvular atrial fibrillation;* SE,* systemic embolism; *VKAs,* vitamin K antagonists

The decision to restore anticoagulants after ICH must balance the patient’s potential risk of thromboembolism and ICH recurrence, which can be measured by CHA2DS2–VASc (congestive heart failure, hypertension, age ≥ 75 years, type 2 diabetes, previous stroke/transient ischemic attack/thromboembolism, vascular disease, age 65 ~ 74 years, and sex category) and HAS–BLED scores (hypertension, abnormal renal/liver function, stroke, bleeding history or predisposition, labile INR, elderly, drugs/alcohol concomitantly) [3]. Concerns for the recurrence of ICH are the main reason for not restarting anticoagulants [127]. The risk factors for recurrent ICH include age, BP, exposure to anticoagulants, concomitant antiplatelet agents, acute or worsening renal failure, the presence or number of multiple CMBs, the mechanism of bleeding (spontaneous versus traumatic), the severity of the bleeding, and the size and location of hematoma, all of which contribute to the individual patient’s risk–benefit assessment of resuming anticoagulants [37, 41, 113, 128]. The analysis of the MGH–ICH study and the ERICH study also suggested that the apolipoprotein E (APOE) ε2/ε4 variants, CMBs and cSS defined by MRI were independently related to ICH recurrence after AAICH [129]. The combination of APOE genotype and MRI markers (cSS and CMBs) helps predict the recurrence of ICH and make clinical decisions in patients with AAICH [129]. In addition, patients with cerebral amyloid angiopathy (CAA) are at higher risk of ICH, so ideally, all patients with CAA and AAICH should avoid anticoagulation, especially long-term anticoagulation therapy [130]. However, RCTs of anticoagulant therapy in patients with CAA have not yet been published. For patients with other risk factors for ICH, such as poorly controlled hypertension, lobar ICH location, and concurrent aspirin use, anticoagulant therapy should be delayed and restarted after achieving BP control or addressing potential risk factors for rebleeding [3, 121, 131]. In patients with non-lobar ICH, the resumption of anticoagulants should be considered depending on the characteristics of bleeding, changes in risk factors, and indications for anticoagulation [21, 37, 41]. While the resumption of anticoagulants should be performed with great caution in those patients with lobar ICH due to the extremely high risk of rebleeding [21, 41]. Once the risk of rebleeding is low enough to no longer exceed the risk of potential recurrent ischemic events, anticoagulation might be further resumed, but it should be noted that the optimal timing of anticoagulation resumption also has an important impact on the outcome of patients with AAICH [132].

Furthermore, when the risk of thromboembolism is extremely high (e.g., mechanical heart valve prosthesis, valvular AF, NVAF with CHA2DS2–VASc score ≥ 4, transient ischemic attack/ischemic stroke within 3 months, VTE within 3 months, or recurrent or cancer-related VTE), resuming anticoagulants may be beneficial to the patients even if the risk of ICH recurrence is high, and should be done as early as possible after hemostasis and stable clinical symptoms [21, 41]. Importantly, for patients at a high risk of thromboembolism and rebleeding who have relative or absolute contraindications to resume anticoagulants (e.g., severe and life-threatening bleeding without treatable causes), nonpharmacological therapy such as left atrial appendage closure/occlusion devices may be considered to alleviate the risk of thrombosis in AF [7, 21, 108, 121]. In addition, anticoagulants are not commonly recommended in patients with CHA2DS2‐VASc scores of 0 to 1 [113, 121]. Moreover, according to European guidelines, individualized treatment decisions are recommended based on the perceived risk of thromboembolism and ICH recurrence [124]. The latest guideline has a class IIB (very weak) recommendation that low-dose UFH or LMWH at 24–48 h from ICH onset may be reasonable and effectively reduce the risk of pulmonary embolism in nonambulatory patients with ICH [7]. Anyway, it is extremely prominent to measure the individual risk factors for thrombosis and bleeding in patients with AAICH.

### Optimal timing of anticoagulation resumption

The clinical decision of when to reinitiate anticoagulants after reversal therapy in AAICH patients remains unclear. The balance between avoiding recurrent ICH and preventing VTE is challenging, especially in the first few days after the onset of ICH. The AHA/ASA guideline in 2015 recommended that anticoagulants should be discontinued for at least 4 weeks after ICH in patients without mechanical heart valves (MHVs) to reduce the risk of recurrent ICH [37]. For patients with a stable ICH and a high risk of cerebral ischemia (e.g., those with mechanical valve prosthesis or NVAF with CHA2DS2VASc score ≥ 4), anticoagulants can be resumed after 14 days [133, 134]. While it may be more appropriate to resume anticoagulants after 4–8 weeks in patients with a moderate or low risk of thromboembolic events [134].

Almost all studies on the evaluation of the optimal timing of anticoagulation resumption after ICH are observational [21]. An observational study identified 137 AAICH patients with MHVs from a nationwide multicenter cohort (RETRACE I and II), showing that the resumption of anticoagulants within less than 2 weeks after ICH in patients with MHV was related to increased hemorrhagic complications. The study weighed the incidence of hemorrhagic and thromboembolic complications and pointed out that the earliest resumption time was at day 6 for patients at high thromboembolic risk (e.g., MHV-patients with concomitant AF, mitral valve prostheses, cage–ball prostheses, etc.) [36]. A retrospective study, including patients with traumatic ICH and subsequently thrombotic complications, showed that therapeutic anticoagulation 1–2 weeks after the onset of ICH was safe when serial CT scans were used to monitor the stability of hematomas [135]. In addition, an observational study suggested that the optimal time was approximately 7–8 weeks after ICH. If anticoagulants were restarted in this interval, it would be beneficial for reducing the risk of thrombotic events, vascular death, and nonfatal stroke without an excess risk of major bleeding [116]. However, some researchers pointed out that this study only showed the safety of warfarin resumption at this time, and the various clinical characteristics, such as CAA or high-risk HE, might affect the timing of anticoagulant resumption [136]. Moreover, the timing for resumption of anticoagulants after ICH was 115 days in the SoSTART trial and 45 days in the APACHE-AF trial [125, 126]. A survey gathered the responses of 163 clinicians and found that 36.6% of clinicians restarted OAC > 30 day post-ICH onset for patients with AF, 24.2% restarted between days 15 and 30 post-event, and 16.3% restarted within the first 10-14 day post-ICH [127]. A recent meta-analysis included 13 studies and showed that the average timing of OAC resumption after ICH was about 30 days [137]. In addition, several RCTs, such as the TIMING trial [138], ELAN trial [139], and the ongoing OPTIMAS trial [140], aimed to explore the optimal timing of anticoagulation after acute ischemic stroke in AF patients. The TIMING trial found that early initiation (≤ 4 days) was not inferior to delayed onset (5-10 days) of DOAC after acute ischemic stroke and early initiation was safe due to the absence of symptomatic ICH [138]. The ELAN trial and a subsequent meta-analysis also suggested that it was safe to start DOAC therapy early compared with delayed initiation [139, 141]. A recent analysis of the PRODAST study included 3312 patients with acute IS or transient ischemic attack (TIA) who were treated with dabigatran or VKA and indicated that the early (≤ 7 days) initiation of dabigatran seemed to be safer and superior than VKA initiated at any time with regards to the risk of ICH [142]. Anyway, the timing of restarting anticoagulation varies greatly amongst centers and regions. There are currently insufficient well-designed randomized trials and sufficiently sized prospective observational studies to explore the optimal timing for the resumption of anticoagulants in patients with AAICH. The timing of anticoagulant resumption should be based on the specific conditions of individual patients [131].

## Conclusions

**Fig. 2:**
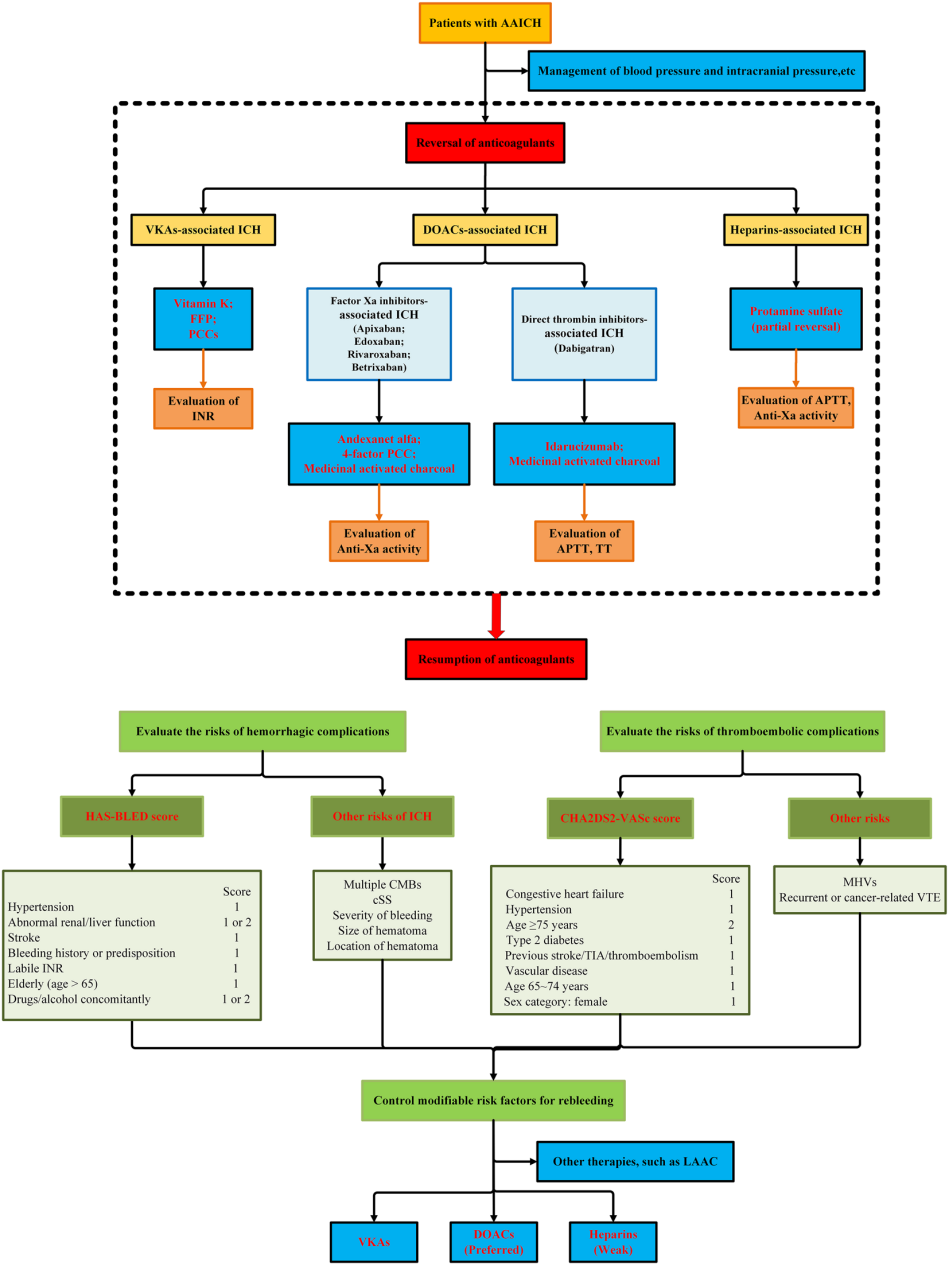
Process of reversal in anticoagulant-associated intracerebral hemorrhage. *AAICH,* anticoagulant-associated intracerebral hemorrhage; *APTT,* activated partial thromboplastin time; *CMBs*, cerebral microbleeds; *cSS,* cortical superficial siderosi; *DOACs,* direct oral anticoagulant; *FFP*, fresh frozen plasma; *ICH,* intracerebral hemorrhage; *INR,* international normalized ratio; *LAAC,* left atrial appendage closure; *MHVs,* mechanical heart valves; *PCC,* prothrombin complex concentrate; *TIA,* transient ischemic attack; *TT,* thrombin time; *VKAs,* vitamin K antagonists; *VTE,* venous thromboembolism.

AAICH is a fatal disease with a poor prognosis, and studies on AAICH are underway. On the one hand, a key point of its management is to stop the use of anticoagulants and reverse coagulopathy. Based on the current literature and guidelines, we have summarized the reversal of anticoagulants in AAICH, and the brief management of coagulopathy in patients with AAICH is presented in Fig. 2. Although significant progress has been made on the reversal of anticoagulants, there is still a lack of strong evidence on the efficacy of new reversal agents, such as ciraparantag and rFVIIa, and on the use of nonspecific reversal agents, such as PCC, in heparin- or DOAC-related ICH. Large and high-quality clinical trials are needed to expand the indications of existing reversal agents and to determine the safety and efficacy of new reversal agents. On the other hand, resuming anticoagulants after AAICH is also an urgent problem affecting the prognosis of patients. In the context of insufficient data from RCTs, the credibility of the evidence for making recommendations is limited. It is necessary to improve the evaluation of coagulation function and weigh the risks of subsequent rebleeding and thromboembolic events in AAICH patients. In conclusion, there is an urgent need for more large-scale RCTs in the future to provide support for the clinical treatment decisions in patients with AAICH.

## Data Availability

All data included in this article are available upon request by contact with the corresponding author.
